# Monolithic Solid Based on Single-Walled Carbon Nanohorns: Preparation, Characterization, and Practical Evaluation as a Sorbent

**DOI:** 10.3390/nano8060370

**Published:** 2018-05-25

**Authors:** Beatriz Fresco-Cala, Ángela I. López-Lorente, Soledad Cárdenas

**Affiliations:** Departamento de Química Analítica, Instituto Universitario de Investigación en Química Fina y Nanoquímica IUNAN, Universidad de Córdoba, Campus de Rabanales, Edificio Marie Curie, E-14071 Córdoba, Spain; q72frcab@uco.es (B.F.-C.); q32loloa@uco.es (A.I.L.-L.)

**Keywords:** carbon monolith, carbon nanoparticles, volatile organic compounds, headspace microextraction, single-walled carbon nanohorns, gas chromatography

## Abstract

A monolithic solid based solely on single walled carbon nanohorns (SWNHs) was prepared without the need of radical initiators or gelators. The procedure involves the preparation of a wet jelly-like system of pristine SWNHs followed by slow drying (48 h) at 25 °C. As a result, a robust and stable porous network was formed due to the interaction between SWNHs not only via π-π and van der Waals interactions, but also via the formation of carbon bonds similar to those observed within dahlia aggregates. Pristine SWNHs and the SWNH monolith were characterized by several techniques, including scanning electron microscopy (SEM), transmission electron microscopy (TEM), confocal laser scanning microscopy, Raman spectroscopy, X-ray photoelectron spectroscopy (XPS), and nitrogen intrusion porosimetry. Taking into account the efficiency of carbon nanoparticles in sorption processes, the potential applicability of the SWNH-monolith in this research field was explored using toluene; m-, p-, and o-xylene; ethylbenzene; and styrene, as target analytes. Detection limits were 0.01 µg·L^−1^ in all cases and the inter-day precision was in the interval 7.4–15.7%. The sorbent performance of the nanostructured monolithic solid was evaluated by extracting the selected compounds from different water samples with recovery values between 81.5% and 116.4%.

## 1. Introduction

The number of publications in the field of nanoscience and nanotechnology has greatly increased in the last years due to the exceptional physical and chemical properties of the nanomatter [1,2,3,4]. Within the sample preparation context, nanoparticles have been extensively used to improve the performance of extractant phases [5,6,7]. In particular, carbon nanostructures have demonstrated a unique sorbent capacity due to their extraordinary surface-volume ratio and the possibility to interact with organic molecules via non-covalent forces. However, most of them are non-porous in nature and possess a high tendency to aggregate when packed as a powder, which requires immobilization on the surface of particles or polymers which are then packed in conventional separation units [8]. These stationary phases present the main problems associated with this type of packing, including the need for frits, bubble formation, and poor mass transfer.

Monolithic solids are porous polymeric networks capable of providing fast mass transport and a high permeability [9]. Many efforts have been devoted to the development of carbon nanoparticle-modified monolithic polymers, which combine the unique and inherent advantages of both materials [10,11,12,13]. Moreover, interconnected porous structures formed in their majority or entirety by carbon nanomaterials are highly desirable [14,15]. These structures can be prepared via chemical vapor deposition (CVD), although a significant amount of residual metal catalyst can be found in the final monolith [16,17]. In addition, wet chemistry methods such as gelation above a critical concentration have been used to create mesoporous materials [18], mainly based on carbon nanotubes. Nevertheless, in most of the cases, extra-linkers (gelation promoters) are needed, as well as the final addition of a structure stabilizer [19,20].

**Scheme 1:**
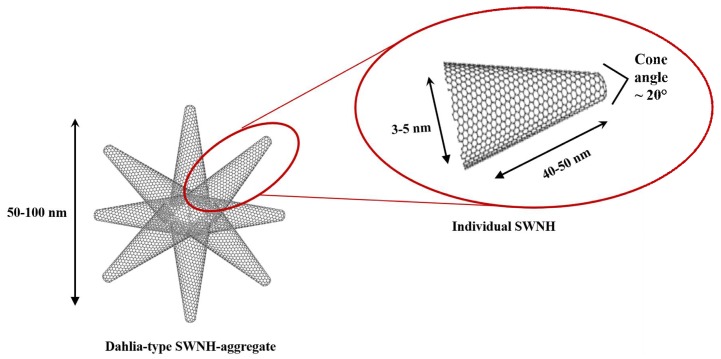
Scheme of dahlia-type aggregates of single walled carbon nanohorns. Detail of an individual single-walled carbon nanohorns (SWNHs) howing its dimensions and the cone angle.

Single walled carbon nanohorns (SWNHs) are carbon nanoparticles characterized by a conical structure with a horn-shaped tip (cone angle of approximately 20°), as well as a cylindrical nanotube section (3–5 nm in diameter and 40–50 nm in length) [21,22]. A feature of SWNHs is their high reactivity, owing to their small size and curvature, which generate an extra negative surface charge, allowing them to react with other radical monomers and initiators [23,24]. In addition, carbon nanohorns are able to form stable aggregates via van der Waals interactions, as well as single C–C bonds between the individual carbon nanohorns [25]. SWNHs have been described to form different types of spherical aggregates, which have been so-called dahlia-, bud-, and seed-type aggregates [26,27]. The resulting spherical dahlia-shaped aggregates (Scheme 1) provide them with an extended surface area and an enhanced sorption capacity in comparison with other carbon nanoparticles such as carbon nanotubes [28]. The inherent tendency of SWNHs to aggregate enables the formation of monoliths without the need for further agents, resulting in a cleaner solid without any impurities from the synthesis.

In this work, we have prepared the first macroscopic SWNH-monolithic solid composed of only dahlia-type aggregates of SWNHs without the assistance of extra components such as dispersants, crosslinkers, or gelation promoters. The gelation is driven by physical interactions including van der Waals forces and π-π stacking. The wet jelly-like system was converted to the corresponding monolithic structure following low rate drying in a polypropylene cell. The solid porous macroscopic structure showed higher stability towards different solvents compared to the monolithic solid composed of only carbon nanotubes. Pristine SWNHs and monoliths were characterized by different techniques, e.g., scanning electron microscopy (SEM), transmission electron microscopy (TEM), nitrogen intrusion porosimetry, Raman spectroscopy, and X-ray photoelectron spectroscopy (XPS), revealing the presence of sp^3^ carbons responsible for the long-term stability of the structure. As a proof of concept, its applicability in the microextraction context has been evaluated for the determination of toluene; ethylbenzene; o-, m-, and p-xylene; and styrene (TEXS) in water samples.

## 2. Materials and Methods

### 2.1. Reagents, Materials, and Samples

All reagents were of analytical grade or better. TEXS (toluene; ethylbenzene; o-, m-, and p-xylene; and styrene) were acquired from Sigma-Aldrich (Madrid, Spain, http://www.sigmaaldrich.com). Individual stock standard solutions (1 g·L^−1^) were prepared in hexane and stored at 4 °C. Working standard solutions were prepared daily by rigorous dilution of the stocks in ultrapure Mill-Q water.

Single-walled carbon nanohorns (SWNHs, >90 wt % purity, 40–50 nm in length and 4–5 nm in diameter) were purchased from Carbonium S.r.l. (Padua, Italy, http://www.carbonium.it/public/site/index.php). SWNHs were dispersed in chloroform (HPLC gradient grade, Sigma-Aldrich, Madrid, Spain) while n-hexane (Panreac, Madrid, Spain) was selected as the eluent.

Tap and Guadalquivir river water samples were selected for the determination of the target compounds following the proposed SWNH-monolithic (micro)solid-phase extraction approach. River waters were collected in amber glass bottles and maintained without headspace at 4 °C until analysis.

### 2.2. Apparatus

Chromatographic equipment was a GC Varian CP-3800 coupled to a Varian 1200 MS/MS working under single quadrupole mode and with an electron multiplier detector. The separation of the six analytes was accomplished on a 5% phenyl-95% dimethylpolysiloxane fused silica capillary column (HP-5MS UI, 30 m × 0.25 mm i.d. × 0.25 µm, Agilent Technologies, Santa Clara, CA, USA). The temperature program was: 40 °C (2 min) initial, raised up to 60 °C at 30 °C·min^−1^ and then immediately ramped at 5 °C·min^−1^ up to 75 °C. The final temperature, 280 °C (1 min), was reached with a ramp of 40 °C·min^−1^. The injector working in splitless mode was kept at 225 °C. The injection volume of n-hexane, 2 µL, was measured with a 5 µL microsyringe (Hamilton Co., Reno, NV, USA). Helium (6.0 grade, Air Liquide, Seville, Spain) was used as the carrier gas at a flow rate of 1.0 mL·min^−1^. It was regulated by a digital pressure and flow controller. The transfer line and ionization source temperatures were 280 °C and 250 °C, respectively.

The ionization mode in the mass spectrometer was electron impact (EI) with an ionization energy of 70 eV. Mass spectra were acquired using the selected ion monitoring mode (SIM), selecting the *m*/*z* 91 at 1 scan·s^−1^. Chromatograms were acquired and processed using the MS Workstation (Varian) on an AMD Sempro™ Processor computer, which also controlled the whole system.

In the preparation of the monolith, a vortex stirrer from IKA^®^ (Staufen, Germany) and an ultrasonic bath (50 W, 40 KHz) form J. P. Selecta (Barcelona, Spain) were used. Besides, an oven (Binder, Madrid, Spain) was also used for drying the monolithic solid at 25 °C.

### 2.3. Preparation of the Monolithic Solid Based on SWNHs

**Figure 1:**
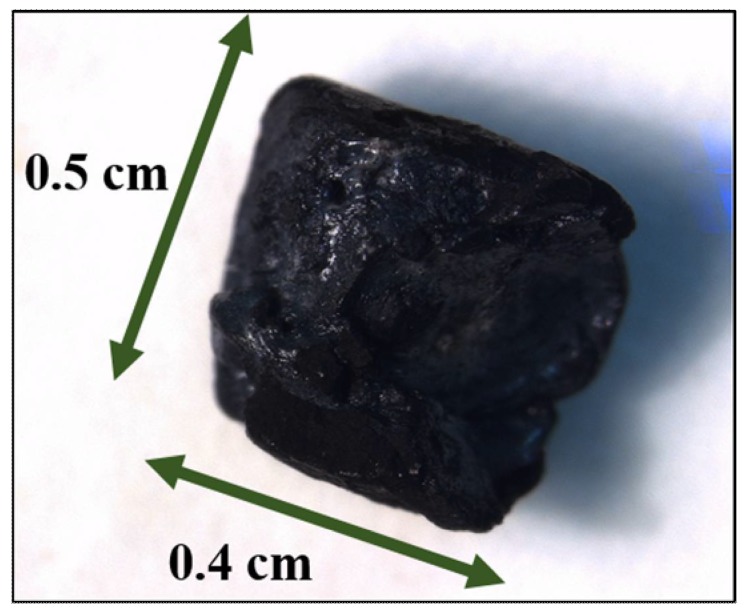
Photograph of the SWNH-monolith.

The macroscopic monoliths were fabricated by crosslinking SWNHs in concentrated suspensions to form wet jelly-like systems. They were prepared by mixing 10 mg of SWNHs and 80 µL of chloroform into a polypropylene device. Next, the mixture was sonicated for 5 min in order to ensure a uniform dispersion of the SWNHs, and then incubated at 25 °C for 48 h. Once formed, the molds were disassembled to obtain the stable macroscopic monolith (Figure 1).

### 2.4. Characterization

Several techniques were used with the goal of characterizing the developed monolith. Raman measurements were carried out with a confocal Raman spectrometer (alpha500, Witec GmbH, Ulm, Germany). A frequency doubled Nd:YAG laser at 532 nm (second harmonic generation) was employed for excitation using a laser power of 2.48 mW (measured prior the objective lens). The laser beam was focused on the sample using a 20×/0.4 Zeiss objective. Raman spectra were acquired with an integration time of 2 s, averaging 10 spectra. For the measurements, solid samples of pristine SWNHs and SWNHs dispersed in chloroform and quickly dried with and without sonication treatment, as well as SWNH-monoliths, were deposited on a glass slide. Measurements were performed at different points of the sample and the average value was calculated. The height of the D and G bands, as well as their ratio, were calculated.

Three-dimensional non-invasive assessment of monolithic surface was performed by confocal microscopy (Leica DCM8, L’Hospitalet de Llobregat, Spain). Surface roughness is given by the average roughness value, Ra, which is the arithmetic mean of the departure of the profile from the center line of a line scan of the surface.

X-ray photoelectron spectroscopy (XPS) was measured with a PHI VersaProbe II spectrometer from Physical Electronics (PHI, Chanhassen, MN, USA), equipped with an Al Kα X-ray radiation source (1486.6 eV). The beam diameter was 200 µm.

A JEOL JSM 7800F scanning electron microscope (Isaza, Alcobendas, Spain) was used to obtain the micrographs of the SWNH-monoliths, and a Philips CM-10 (Philips, Amsterdam, Netherlands) transmission electron microscope (TEM) was used to obtain the micrographs of the SWNHs’ dahlia-like structure.

Nitrogen adsorption/desorption experiments were carried out at −196 °C using Quantachrome^®^ ASiQwin^TM^-Automated Gas Sorption Data. The specific surface area values were calculated according to the BET (Brunauer-Emmett-Teller) equation. The average pore diameter and volumes were evaluated from the desorption branches of isotherms based on the BJH (Barrett-Joyner-Halenda) model.

## 3. Results and Discussion

### 3.1. Synthesis of SWNH-Monolith

The formation of pure carbon nanohorn monoliths has been deeply studied in this work. The main objective was the fabrication of a monolithic solid based on pristine SWNHs maintaining the unique characteristics and properties related with their shape and size. For that purpose, a given amount of SWNHs was dispersed in an organic solvent (chloroform) and sonicated for 5 min. Above a critical concentration, the mixture formed a slightly sticky system due to van der Waals and π-π interactions. At high concentrations, the distance between the dahlias aggregates is reduced, inducing SWNH interactions. In fact, higher concentrations lead to denser and mesoporous structures, while if the concentration is too low, only a precipitate is formed [19]. Thus, pure SWNH systems were obtained at a concentration of 125 mg·mL^−1^, allowing the complete removal of the solvent by drying at 25 °C without destroying the monolith.

Usually, radical initiators are used for the covalent functionalization of carbon nanostructures [29], as well as for the fabrication of macroscopic stable carbon scaffolds [30], while the use of linkers or initiators helps to obtain interlinked solids with excellent mechanical resilience. Herein, the fabrication of mechanically stable carbon nanohorns without the need for radical initiators has been possible thanks to the enhanced chemistry reactivity of SWNHs and their tendency to form stable aggregates. The stability of the SWNH-monoliths was evaluated by their immersion in different solvents including water, methanol, and hexane (see Figure S1), and via submission to high temperatures, in order to evaluate their practical stability for future applications. In all cases, the monolith exhibited great stability, maintaining its shape and rigidity. Moreover, once the monolith structure was formed, the structures were manipulated with the aid of tweezers for the subsequent microextraction procedure. It has to be pointed out that the solid monolithic structure was not damaged by manual handling with the tweezers during the different steps of the extraction process.

### 3.2. Microscopic and Pore Characterization of SWNH-Monoliths

As previously mentioned, SWNHs possess a conical structure with a horn-shaped tip and a cylindrical nanotube section (~2–5 nm in diameter and 40–50 nm in length). The conical shape of carbon nanohorns is the direct consequence of introducing six pentagons within the network of hexagons of the graphene sheet pentagons. An additional heptagon is also required to counteract the curvature change of one pentagon.

**Figure 2:**
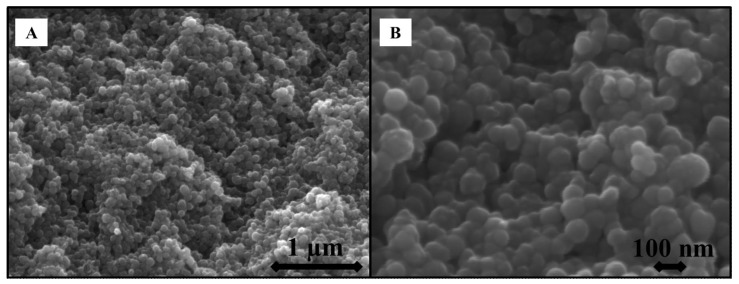
Scanning electron microscopy (SEM) of the SWNH-monolithic solid at 27,000× (**A**), and 70,000× (**B**) magnification.

**Figure 3:**
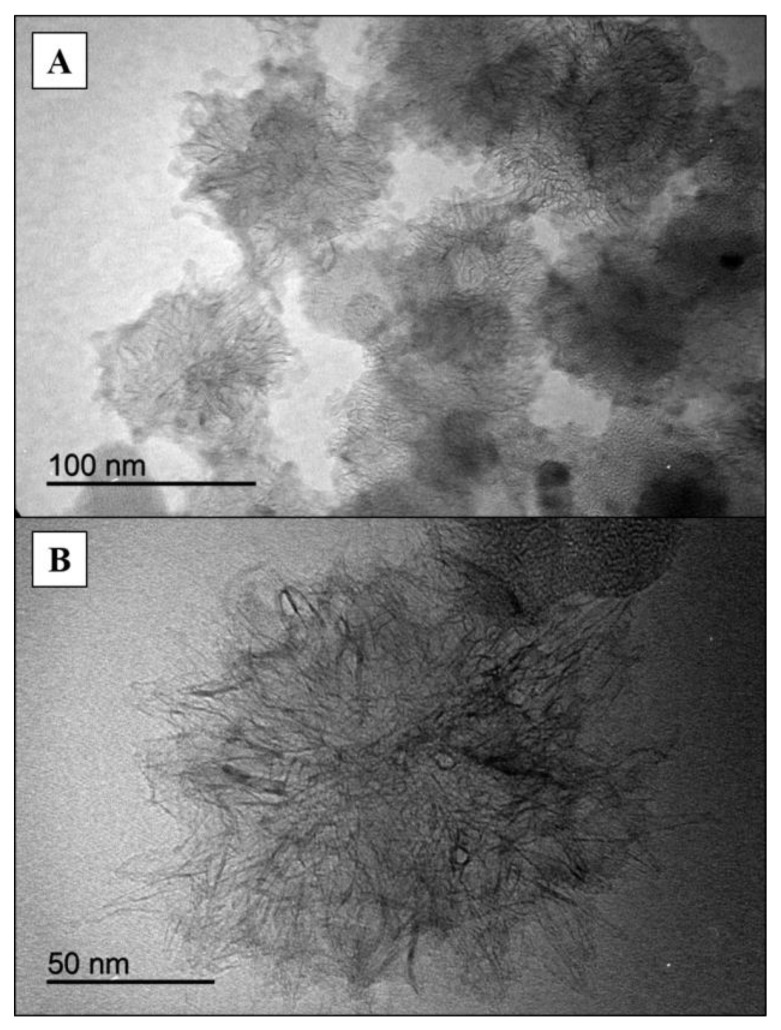
Transmission electron microscopy images of SWNHs at low (**A**) and high (**B**) magnification.

SEM images of the SWNH-monoliths were acquired to characterize their morphology and porous structure (Figure 2). From the microphotographs at a 27,000× magnification (Figure 2A), the monolithic solid clearly exhibited interconnected SWNHs which results in a porous architecture. At a larger magnification (70,000×, Figure 2B) of the globules, the image showed that the size of globules varies between 50–100 nm, which is coherent with the diameter of the spherical dahlia-shaped aggregates. This fact was corroborated by the TEM micrographs acquired from pristine SWNH dahlia aggregates (Figure 3), which also showed that the nanoparticles are aggregated into dahlia-type structures of a nearly spherical shape, showing a narrow size distribution (Figure 3A). A magnified TEM micrograph of an SWNH dahlia-aggregate (Figure 3B) showed conical horn-like protrusions in the edge area, corresponding to the individual SWNHs.

**Figure 4:**
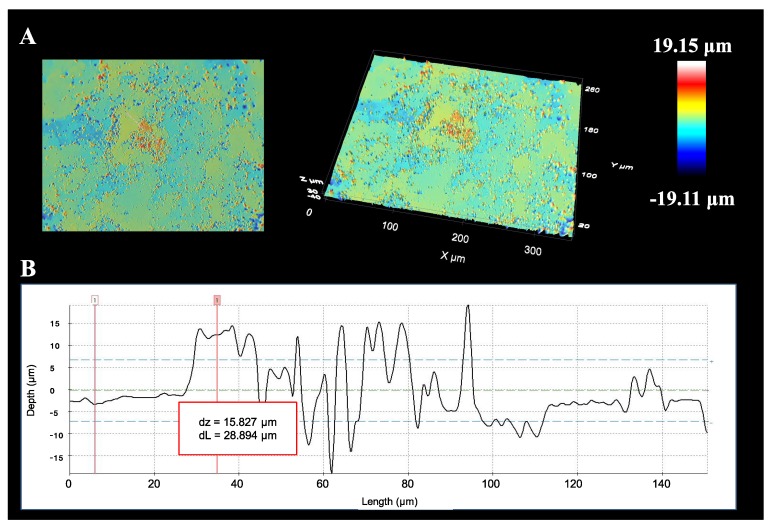
2D and 3D images of the SWNH-monolith (**A**), and its depth profile (**B**) in confocal microscopy.

The confocal profile image of the SWNH-monolith is shown in Figure 4. 2D and 3D representations of their surface topographies with a color scale for the z-range calibrated in micrometers (µm) are illustrated. It is possible to distinguish the surface roughness of the macroscopic SWNH-monolithic solid as well as the depth profile along a vertical line with a mean of the absolute values of the profile heights measured from a mean line averaged over the profile (Ra) of 3.685 μm.

**Figure 5:**
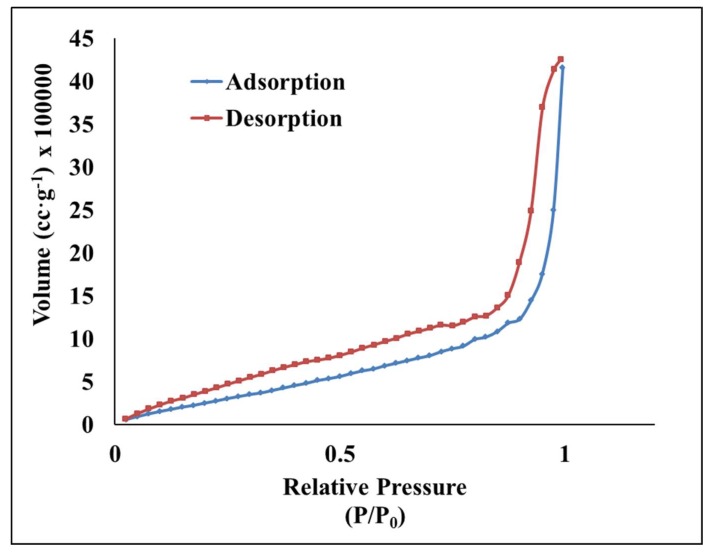
Nitrogen adsorption and desorption isotherms of the SWNH-monolith.

Nitrogen intrusion porosimetry measurements were performed to assess the porosity of SWNH-monoliths. The textural values including the specific surface area, pore diameter, and pore volume, are compiled in Table 1. Data from N_2_ adsorption-desorption isotherms evidenced that the solid exhibited an isotherm type IV with a step at a relative pressure around 0.7–0.9 (Figure 5). This is typical of pores with sizes in the high mesopore range.

### 3.3. Spectroscopic Characterization of SWNH-Monoliths

**Figure 6:**
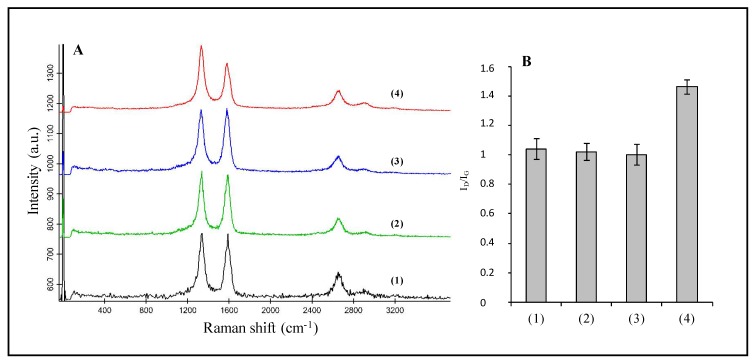
(**A**) Raman spectra of pristine SWNHs (1), SWNHs dispersed in chloroform followed by a rapid evaporation of the solvent without sonication (2) and with a previous sonication step (3), and the SWNH-monolith (4). (**B**) Plot of the average value of the intensity ratio of the Raman D and G bands calculated from the Raman spectra measured of the different materials. Spectra at different positions within each sample were acquired and the average value calculated (n = 5), with the error bars depicting the standard deviation within measurements.

Moreover, the synthesized SWNH-based monoliths and pristine SWNHs were also characterized by different spectroscopic techniques, i.e., Raman spectroscopy and X-ray photoelectron spectroscopy. Figure 6A shows the Raman spectra of pristine SWNHs (1), that of SWNHs dispersed in chloroform followed by a rapid evaporation of the solvent with (3) and without (2) the previous sonication step, and the SWNH-monolith prepared as previously described (4). As can be seen, the Raman spectrum of the pristine SWNHs at room conditions exhibits the characteristic bands typical of sp^2^ defected carbon materials, i.e., the G band (~1589 cm^−1^) associated with the double bonds between the carbon atoms (C=C), which originates from in-plane E2g stretching vibrations in the sp^2^ bonded carbon atoms [31], and the D band (~1350 cm^−1^), assigned to A1g-symmetry modes [32], which is related to the introduction of pentagons and heptagons resulting in the typical conical-shape of SWNHs, as well as with the presence of single-bonding sp^3^ carbon atoms (C–C) [26,33].

The spectrum of the SWNH-monolith material is also dominated by these two characteristic bands; nevertheless, an increase in the D feature as regards the G band is observed compared to the pristine material. The intensity ratio of the D and G bands was calculated for the different materials. As shown in Figure 6B, the intensity ratio I_D_/I_G_ increases from 1.04 for pristine SWNHs to 1.46 for the SWNH-monolith. The D band is associated with structure imperfections/defects, thus, the effect of the sonication process on the structure of the SWNHs was evaluated. For this purpose, solid SWNHs were dispersed in chloroform and a rapid evaporation process was carried out without previous sonication of the solid (spectrum 2 in Figure 6A). On the other hand, SWNHs dispersed in chloroform were submitted to the same sonication conditions as those of the monolith, with the drying step taking place in a short time (spectrum 3, Figure 6A). As can be seen in the spectra and deduced from the calculated I_D_/I_G_ values, sonication of the nanomaterial at such conditions does not lead to the introduction of defects within the SWNHs’ structure and, thus, to the observed variations in the Raman bands.

**Figure 7:**
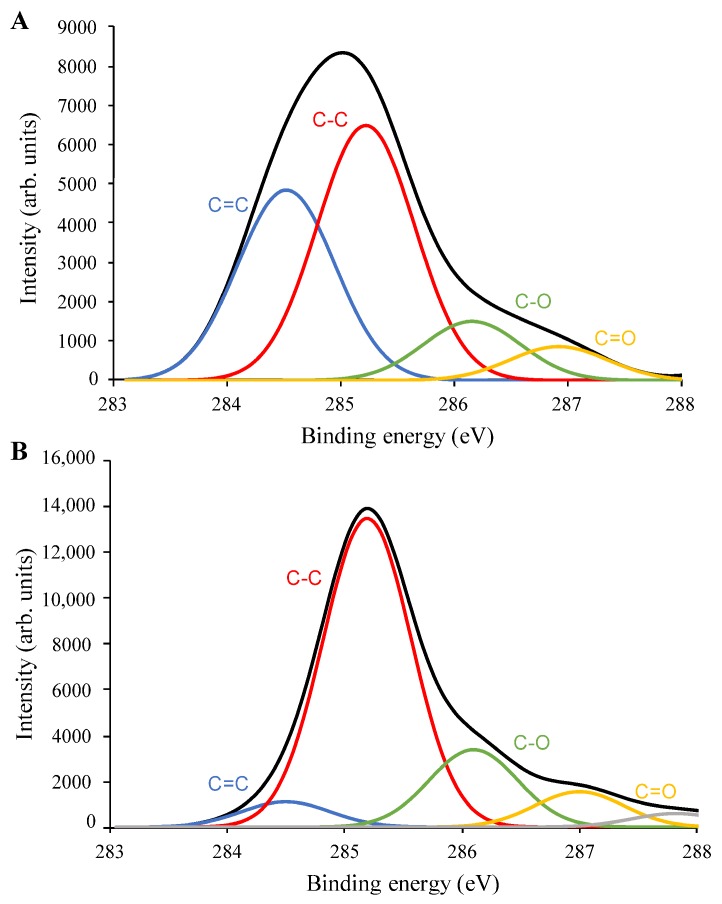
X-ray photoelectron spectroscopy C1s spectra and deconvolution results of (**A**) pristine SWNHs, and (**B**) SWNH-monolith.

A feature of SWNHs is that they are hardly dispersed into individual primary particles, even under sonication in surfactant solutions. This characteristic contrasts with that of other carbon nanostructures, such as single walled carbon nanotubes (SWCNTs), which can be easily dispersed into individual tubes, thus leading to the hypothesis that SWNH aggregation is owing not only to van der Waals interactions, but also chemical bonding, which may contribute to the stability of such aggregates [25]. As reported by Utsumi et al. [25], C–C single bonds between dahlia-aggregates contribute to the assembly structure, which is reflected by a strong D-band in the Raman spectrum. Thus, the high stability of the macroscopic monolithic solid may be the result of the formation of sp^3^ single C–C bonds between aggregates, as reflected by an increase in the I_D_/I_G_ ratio (Table 2), which is in agreement with the C=C and C–C bonds ratio determined by XPS (Figure 7). The relevance of the slow rate of the drying process at the polypropylene device should be pointed out, which enables the formation of bonds between aggregates. Interestingly, monolithic solids created from carbon nanotubes did not show the same stability towards water and solvents, the Raman spectra of the solid CNTs (Figure S6A), and the macroscopic tridimensional structure (Figure S6B), revealing a similar I_D_/I_G_ ratio compared to the powder of CNTs, as shown in the Table 2.

Figure 7 shows the C1s XPS spectra of pristine SWNHs and the SWNH-monolith. The C1s spectrum of pristine SWNHs shows a relatively broad peak with the maximum at about 285 eV, while the C1s spectrum of the SWNH-monolith presents a narrower band centered at about 285.2 eV. The deconvoluted C1s spectrum of pristine SWNHs (Figure 7A) shows two main contributions at about 284.5 eV and 285.2 eV. The peak at 284.5 eV is assigned to the double bonding carbons (C=C) corresponding to carbons with sp^2^ hybridization typical of SWNHs [25]. On the other hand, the sub-peak at 285.2 eV can be assigned to single bonding carbons (C–C) of carbons displaying sp^3^ hybridization [25]. Other sub-peaks are also observed at a higher binding energy, which can be ascribed to oxygen-containing functional groups, such as C–O at 286.1 eV and C=O at 287.0 eV. Figure 3B depicts the deconvoluted C1s XPS spectrum of the SWNH-monolith. As can be seen, the narrower band centered at about 285.2 eV has a major contribution of the C–C bonding (sp^3^ hybridization), while the contribution of the sub-peak at 284.5 eV corresponding to the sp^2^ carbon has significantly decreased compared sp^3^ carbon. This increase in the C–C bonds as regards C=C is in agreement with the increase in the D band value observed in the Raman spectrum of the SWNH-monolith. According to Kawai et al. [34], in junction structures within graphene sheets or tubes, the carbons may acquire sp^3^ hybridization, which has been associated with the firm structure of SWNH aggregates.

### 3.4. Microextraction Procedure

The microextraction procedure for the extraction of TEXS from water samples is described as follows. Aliquots of 50 mL of aqueous standards or water samples were placed in a 100 mL glass beaker and a magnetic stirrer was added. Next, the SWNHs monolith supported on a rubber platform fixed to a stainless-steel wire, was exposed to the sample headspace. Then, the standard/sample was magnetically stirred (15 min, 275 rpm) at room temperature. Next, analytes were eluted by direct immersion of the SWNH-monolith, enriched with the analytes, in a glass insert containing 200 μL of n-hexane (5 min, vortex agitation). Volumes of 2 μL of the final hexane phase with the extracted analytes were injected into the GC/MS for separation and detection. Between samples, the solid was washed with n-hexane and then dried in an oven at 100 °C.

The selection of the most favorable values for all the variables affecting the microextraction procedure was assessed by means of a univariate model. In this way, those variables having a major influence on the process could be clearly identified. For this aim, aqueous standards containing the six volatile analytes at a concentration of 10 µg·L^−1^ were prepared. The initial conditions of the extraction were: 25 mL of aqueous standard, and magnetic stirring during 20 min at 275 rpm at a temperature of 25 °C using 200 μL of n-hexane as the eluent.

Several parameters were optimized, namely: (a) sample volume; (b) stirring rate; (c) extraction time; and (d) extraction temperature, and the results are explained in detail in the Electronic Supporting Information. The optimum values were: (a) sample volume of 50 mL; (b) stirring rate of 275 rpm; (c) extraction time of 15 min; and (d) extraction temperature of 90 °C.

### 3.5. Analytical Figures of Merit and Analysis of Water Samples

The headspace microextraction method, working under the selected experimental conditions, was characterized in terms of a linear interval, limits of detection and quantification, and precision. The values are summarized in Table 3. Calibration curves were calculated using nine working aqueous standards, with each one prepared in duplicate. The corresponding equations were obtained by plotting the peak areas of the characteristic m/z fragment ions versus the concentration for each target analyte and were linear from 0.1 to 10,000 µg·L^−1^. The limits of detection (LODs) and quantification (LOQs) were obtained using a signal-to-noise ratio (S/N) of 3 and 10, respectively. The calculated values were 0.01 µg·L^−1^ (LOD) and 0.1 µg·L^−1^ (LOQ) for all analytes. The precision of the method was studied under repeatability and reproducibility experimental conditions. Repeatability (intra-day conditions), expressed as relative standard deviation (RSD), was lower than 6.88% (n = 5) for all the analytes. The reproducibility between days (inter-day conditions) ranged from 7.37% to 15.7% (n = 5). The enrichment factors for all the analytes were calculated by a comparison of the slopes of the calibration graphs before and after the extraction process. They were in the range from 11.3 to 26.9.

The proposed method was applied to the determination of TEXS in environmental waters (tap and river). The samples were analyzed under the optimized conditions. Since none of the analytes were detected, blank tap and river waters were enriched with the six target analytes at a concentration of 1 µg·L^−1^. The concentrations were calculated by interpolating the peak area obtained in the corresponding calibration graph. The recovery values (average of three replicates calculated dividing the concentration found by the concentration added, expressed in percentage) are shown in Table 4. They ranged from 85.9% to 116.4% and 81.5% to 89.8% for tap and river waters, respectively.

Table S2 compares the performance of the proposed microextraction method with that provided by recent approaches developed for the same analytical problem [35,36,37,38,39]. As it can be seen, the SWNH-monolith provides equal or better sensitivity (PDMS-grafted carbon nanospheres excepted) with comparable extraction recoveries. As an advantage, it allows processing large sample volumes in an intermediate time interval as regards fiber-based extraction. Regarding the method based on aerogels, the extraction capacity of the SWNH-monolith is higher, which results in better detection limits.

## 4. Conclusions

A monolith based on carbon nanohorns has been synthesized for the first time. The procedure is quite simple as it only requires the preparation of a stable dispersion of pristine SWNHs in chloroform using ultrasound energy. The dispersion is placed in a polypropylene device with the desired geometry and left to stand for slow solvent evaporation for 48 h at 25 °C. The monolith thus obtained is hydrophobic in nature and stable in organic solvents. As an advantage over other synthetic procedures described in the literature for similar porous structures, no crosslinker or initiators (gelation promoters) are required. This results in a cleaner solid without any impurities from the synthesis. The solid was characterized by several techniques including SEM, confocal laser scanning microscopy, Raman spectroscopy, XPS, and nitrogen intrusion porosimetry, which proved the increase in sp^3^ bonds within the monolithic material as regards pristine SWNHs, which accounts for the high stability of the porous macrostructure as regards other carbon nanomaterials with just van der Waals and π-π intermolecular interactions. Finally, the material has been evaluated in the microextraction context using toluene; m-, p-, and o-xylene; ethylbenzene; and styrene as model analytes. The excellent results obtained envisage an extended applicability of the carbon nanoparticles, and in particular of SWNHs, within the analytical field.

## Figures and Tables

**Table 1:** Porous properties of the SWNHs monolithic solid.

Specific Surface Area, BET (m^2^·g^−1^)	Average Pore Diameter (Å)	Average Pore Volume (cc·g^−1^)
154.68	166	0.64

**Table 2:** Ratio of the intensity of the D and G band from the Raman spectra of the pristine carbon nanomaterials, namely carbon nanotubes (CNTs) and single-walled carbon nanohorns (SWNHs) and their corresponding monoliths.

	Pristine CNTs	CNT-Monolith	Pristine SWNHs	SWNH-Monolith
I_D_/I_G_ ratio	0.90 ± 0.09	0.90 ± 0.07	1.04 ± 0.07	1.46 ± 0.05

**Table 3:** Analytical figures of merit of macroscopic 3D SWNHs monoliths for the determination of the target TEXS.

Analyte	*m*/*z*	LOD (µg·L^−1^)	LOQ (µg·L^−1^)	Precision		EF
				Intra-Day RSD (%, n = 5)	Inter-Days RSD (%, n = 5)	
Toluene	91	0.01	0.1	3.3	10.8	15.0
Ethylbenzene	91	0.01	0.1	4.9	14.2	26.9
m- and p-Xylene	91	0.01	0.1	6.2	8.4	11.3
o-Xylene	91	0.01	0.1	6.5	15.7	22.6
Styrene	104	0.01	0.1	6.9	7.4	16.3

LOD limit of detection, LOQ limit of quantification, RSD relative standard deviation, EF enrichment factor.

**Table 4:** Recovery study for the target TEXS spiked to water samples analyzed using the SWNHs monolith.

Analyte	Tap Water (%, n = 3)	River Water (%, n = 3)
Toluene	93 ± 6	90 ± 7
Ethylbenzene	87 ± 3	82 ± 16
m- and p-Xylene	116 ± 13	84 ± 14
o-Xylene	91 ± 5	81 ± 16
Styrene	86 ± 8	85 ± 15

## Supplementary Materials

The following are available online at http://www.mdpi.com/2079-4991/8/6/370/s1, Table S1: Variables studied in the headspace microextraction indicating the initial value, the interval studied, and the selected value, Figure S1: Photographs of the solid monoliths after 2 h immersion in different solvents, namely water (a), methanol (b) and hexane (c), Figure S2: Effect of the sample volume on the analytical signal obtained after the microextraction procedure, Figure S3: Effect of the stirring rate on the analytical signal obtained after the microextraction procedure, Figure S4: Effect of the extraction time on the analytical signal obtained after the microextraction procedure, Figure S5: Effect of the extraction temperature on the analytical signal obtained after the microextraction procedure, Figure S6: Raman spectra of pristine CNTs (A), and CNT-monolith (B), Table S2: An overview on recently reported headspace methods for preconcentration and determination of BTEXs.
