# Supplementary material for: Monolithic Solid Based on Single-Walled Carbon Nanohorns: Preparation, Characterization, and Practical Evaluation as a Sorbent
Source: Nanomaterials (Basel). 2018 May 25;8(6):370. doi: 10.3390/nano8060370 (PMC6027447; doi:10.3390/nano8060370)
Supplement: Supplementary file 1 [file nanomaterials-08-00370-s001.pdf]

Electronic Supplementary Material

Monolithic Solid Based on Single-Walled Carbon Nanohorns: Preparation, Characterization, and Practical Evaluation as a Sorbent

Beatriz Fresco-Cala[1], *Ángela I. López-Lorente*[1] and *Soledad Cárdenas* [1*]

[1] *Departamento de Química Analítica, Instituto Universitario de Investigación en Química Fina y Nanoquímica IUNAN, Universidad de Córdoba, Campus de Rabanales, Edificio Marie Curie, E-14071 Córdoba, España.*

* Correspondence: scardenas@uco.es; Tel.: +34 957218616

1. Stability of monoliths towards solvents

The stability of the SWNH-monoliths towards solvents was evaluated by their immersion in different solvents including water, methanol, and hexane for two hours. As shown in Figure S1, the monoliths remained stable when using different solvents.

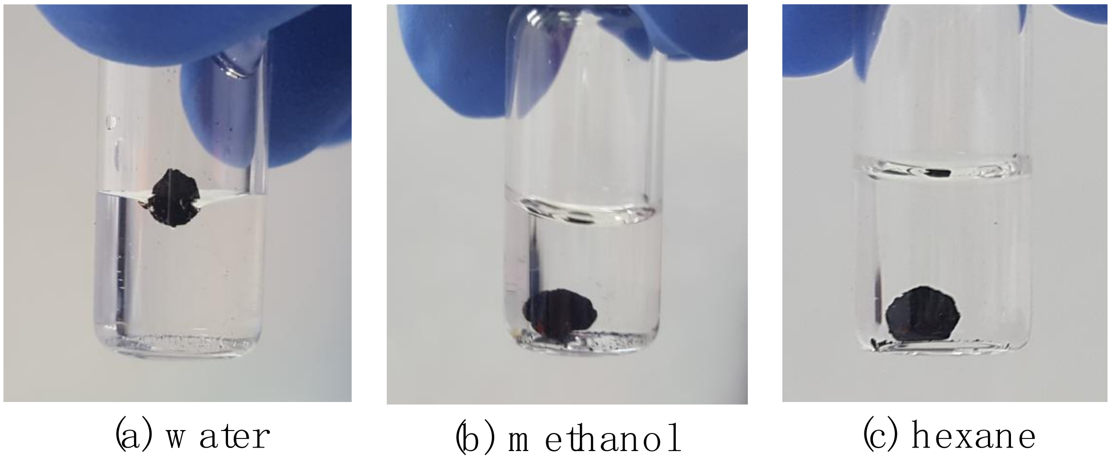

Figure S1. Photographs of the solid monoliths after 2 h immersion in different solvents, namely water (a), methanol (b), and hexane (c).

2. Variables affecting the microextraction procedure

Different variables may affect the efficiency of the microextraction procedure and therefore their effect on the analytes extraction was considered in depth. Table S1 reflects their initial values, the interval studied, and the optimum values for each variable. The optimization was performed under a univariate approach using an aqueous standard solution containing the six analytes at a concentration of 10 $\mu g \cdot L^{-1}$.

Table S1. Variables studied in the headspace microextraction indicating the initial value, the interval studied, and the selected value.

Variable	Initial value	Interval studied	Selected value
Sample volume (mL)	25	10-50	50
Stirring rate (rpm)	275	0-550	275
Extraction time (min)	20	10-30	15
Extraction temperature (°C)	25	25-90	90

The sample volume was studied in the interval 10-50 mL, keeping the analytes concentration constant. As it is shown in Figure S2, the analytical signal obtained for the target analytes increases up to 50 mL, and therefore it was found to be the most convenient value.

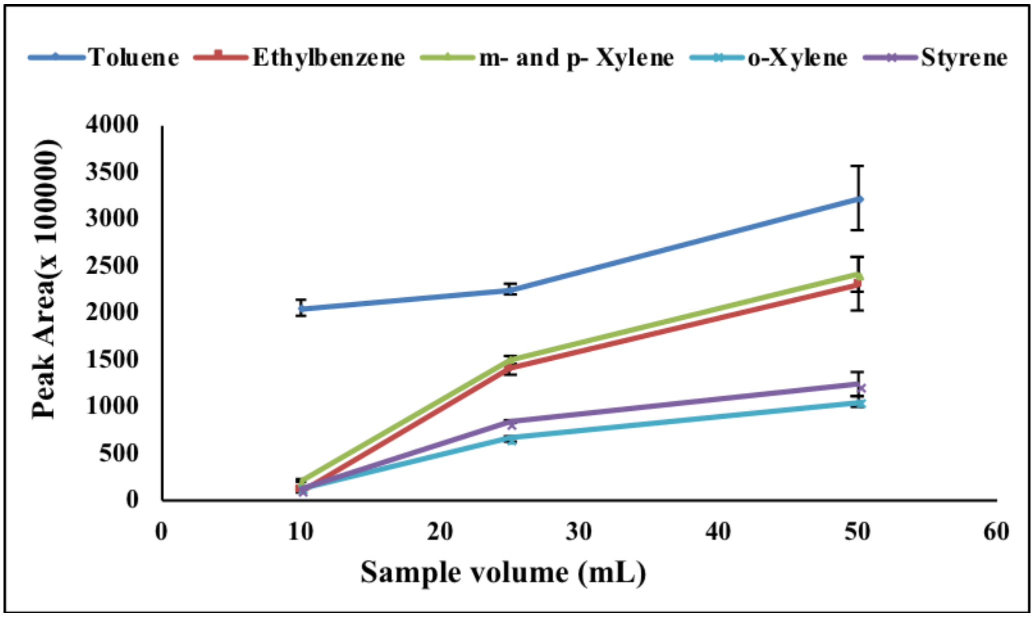

Figure S2. Effect of the sample volume on the analytical signal obtained after the microextraction procedure.

The next variable evaluated was the stirring rate of the sample within the interval 0-550 rpm. As it can be seen in Figure S3, the peak areas increased up to 225 rpm, reaching an almost steady state over this value.

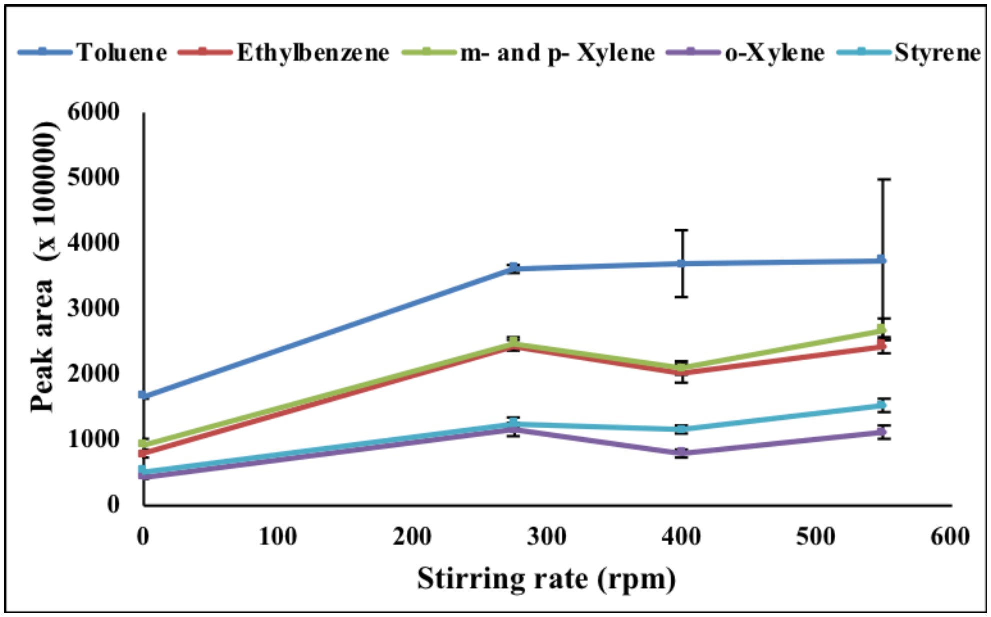

Figure S3. Effect of the stirring rate on the analytical signal obtained after the microextraction procedure.

Concerning the extraction time, this variable was evaluated between 10 and 30 min (Figure S4). The results pointed out that this variable positively affects the extraction up to 15 min, decreasing over this value.

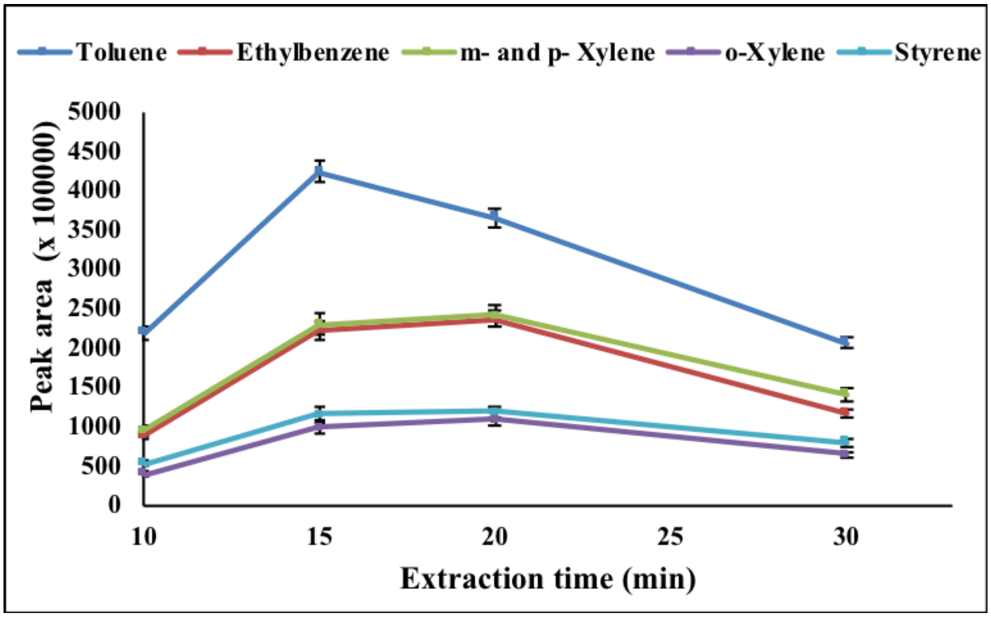

Figure S4. Effect of the extraction time on the analytical signal obtained after the microextraction procedure.

Finally, the influence of extraction temperature during the extraction was evaluated from 25 to 90 °C (Figure S5). An increase in the temperature facilitates the release of these analytes to the headspace of the vial and thus the adsorption at the surface of the SWNH-monolith. Temperatures higher than 90 °C were not evaluated considering the aqueous nature of the standard solutions and further application to water samples.

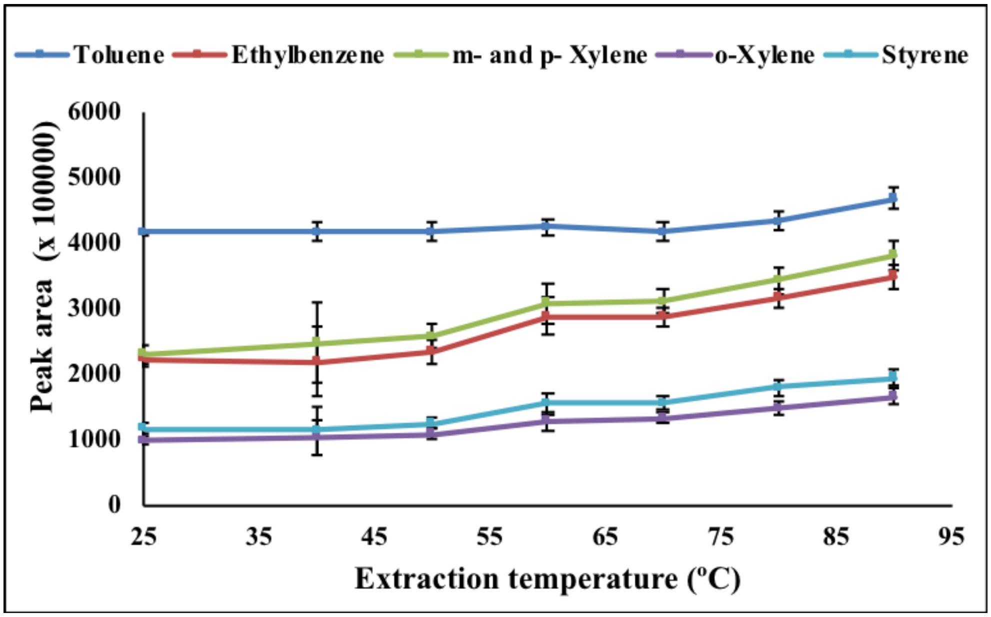

Figure S5. Effect of the extraction temperature on the analytical signal obtained after the microextraction procedure.

3. Raman spectroscopy characterization

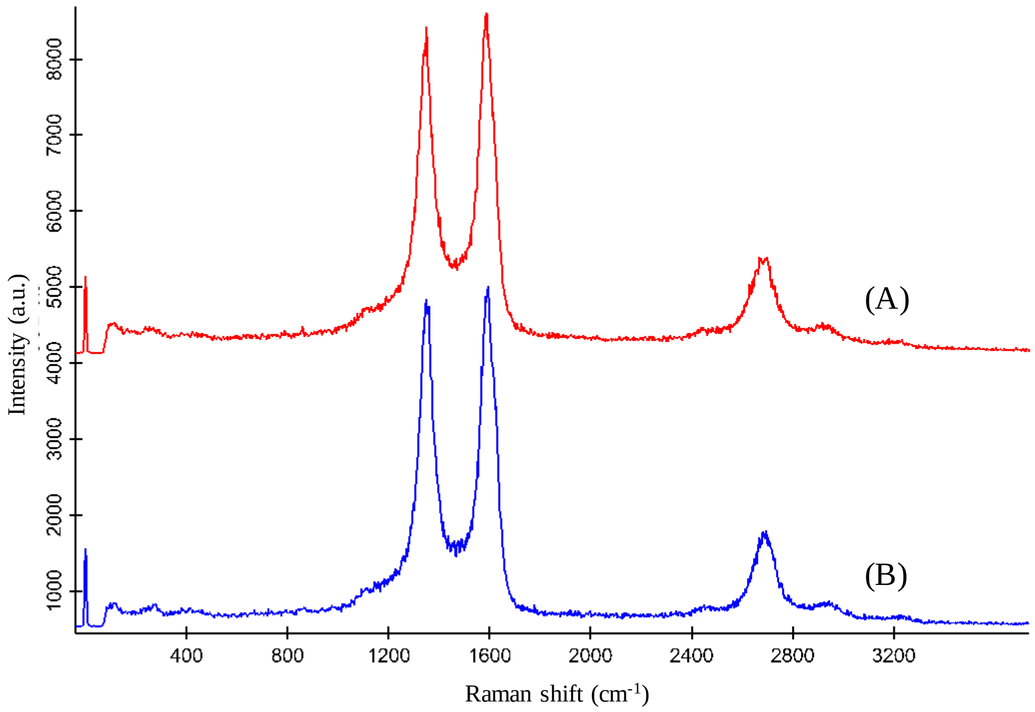

Figure S6. Raman spectra of pristine CNTs (A), and the CNT-monolith (B).

Table S2. An overview of recently reported headspace methods for the preconcentration and determination of BTEXs.

Material used	Sample	Sample volume (mL)	Extraction time (min)	Detection method	LODs ($\mu g \cdot L^{-1}$)	LOQs ($\mu g \cdot L^{-1}$)	R (%)	Ref
PDMS-grafted carbon nanospheres	Tap water, river water, well water and wastewater	15	25	GC-FID	0.001-0.01	0.003-50	92.5-99.5	[28]
Polypyrrole-carbon nanotubes-titanium oxide (PPy-CNT-TiO_2)	Tap water, mineral water, river water, well water and wastewater	15	35	GC-FID	0.01-0.04	0.03-500	93.7-106.2	[29]
Poly(o-anisidine)/ graphene oxide nanosheets composite (PoA/GONSs)	River and agricultural well water	10	30	GC-MS	0.01-0.06	0.1-500	92.0-101.2	[30]
Metal-organic frameworks (MIL-101(Cr))	River water	10	3	GC-MS	0.32-1.7	10-20000	80.0-1130.0	[31]
Powdery polymer aerogel- carbon aerogel (PPA-PCA)	River, pond and tap water	-	7-9	GC-MS	0.2-0.9	5-5000	82.9-102.0	[32]
SWNHs monolith	Tap and river water	50	15	GC-MS	0.01	0.1-10000	81.5-116.4	This work

LOD limit of detection, LOQ limit of quantification, R recovery values, Ref references.
